# Chromosomal aberrations in human lymphocytes and fibroblasts after exposure to very low doses of high-LET radiation

**DOI:** 10.1093/jrr/rrt212

**Published:** 2014-03

**Authors:** Megumi Hada, Kerry George, Lori Chappell, Francis A. Cucinotta

**Affiliations:** 1 Universities Space Research Association, Houston, USA; 2 Wyle Science, Technology, & Engineering Group, Houston, USA; 3 NASA Lyndon B. Johnson Space Center, Houston, USA; 4 University of Nevada, Las Vegas, Las Vegas, NV 89154, USA

**Keywords:** low dose, heavy ion, chromosome aberrations

## Abstract

**Purpose:** The relationship between biological effects and low doses of radiation is still uncertain, especially for high-LET radiation exposures. Estimates of risk from exposure to low doses and low dose rates are often extrapolated from the Japanese atomic bomb survivor data using either linear or linear-quadratic models fitted to dose–response data. In this study, we determined the dose–response for chromosome damage after exposure to very low doses of high-LET radiation and assessed the radiation qualities of Fe, Si and Oxygen ions.

**Materials and methods:** Chromosomal aberrations (CA) were measured in human peripheral blood lymphocytes and normal skin fibroblasts after exposure to very low doses (0.01–0.20 Gy) of 77-MeV/u oxygen (LET = 55 keV/µm), 170-MeV/u ^28^Si (LET = 99 keV/µm), or ^56^Fe ions with energies of 600- or 450-MeV/u (LET = 180 or 195 keV/µm). These exposures included doses that, on average, produce fewer than one in five direct ion traversals per cell nucleus. Chromosomes were analyzed using the whole-chromosome fluorescence *in situ* hybridization (FISH) technique during the first cell division after irradiation, and CA were identified as either simple exchanges (translocations and dicentrics) or complex exchanges (involving more than two breaks in two or more chromosomes). The frequencies of CA in the painted chromosome(s) were evaluated as the ratio between aberrations scored and total cells analyzed. The dose–response for simple exchanges was assessed using a generalized linear model assuming binomial errors per number of chromosomes scored. The model coefficients were extrapolated to whole-genome equivalents. The linear dose–response denoted as the targete effects (TE) model considered the mean number of radiation tracks per cell. Two different non-targeted effect (NTE) models, *P* = *P*_0_ + *αT* + κ × *I* (NTE1), and *P* = *P*_0_ + *αT* (1 − *e*^−T^) + *κe*^−T^ × *I* (NTE2), were compared with the simple linear model, *P* = *P*_0_ + *αT*. Akaike information criteria (AIC) and Bayes information criteria (BIC) were used to compare TE and NTE models for fitting chromosome aberrations in low dose range.

**Results:** Doses that on average produce more than one ion traversal per cell nucleus showed a linear dose–response for CA in both lymphocytes and fibroblasts. However, for doses that produce fewer than one tracks per cell in fibroblasts, O, Si and Fe particles showed a dose-independent response for CA that was significantly elevated relative to background frequencies. For fibroblasts the NTE model 2, *P* = *P*_0_ + *αT* (1 − *e*^−T^) + *κe*^−T^ × *I*, showed improved fit to CA in low dose range compared with TE model or NTE1 model. For lymphocytes, tests of the various models were less clear with TE model optimal for Si and Fe while the NTE2 model optimal for O particles. When low-dose exposures were fractionated with 2-h intervals, increased frequencies of both simple and complex exchanges were observed. Nitric oxide scavenger reduced CA induced by low doses of high-LET irradiation. Inhibition of transforming growth factor-β receptor-1 reduced the frequency of simple exchanges.

**Conclusions:** The results show a non-linear dose–response for CA in fibroblasts after very low doses of high-LET exposure. Possible explanations for this could involve non-targeted effects due to aberrant cell signaling [ 1], perhaps involving nitric oxide and TGF-β, or could be due to delta-ray dose fluctuations [ 2] where CA are induced in cells that receive a significant dose from delta-rays emanating from the multiple ion tracks that do not directly traverse cell nuclei.
